# Synthesis of 9,9′-[1,2-Ethanediylbis(oxymethylene)]bis-2-amino-1,9-dihydro-6*H*-purin-6-one, an Impurity of Acyclovir

**DOI:** 10.3390/molecules17088735

**Published:** 2012-07-25

**Authors:** Rosa M. Suárez, Maria Paz Matía, José Luis Novella, Andres Molina, Antonio Cosme, Juan José Vaquero, Julio Alvarez-Builla

**Affiliations:** 1 Fine Chemicals Pilot Plant Facility, University of Alcalá, 28871 Alcalá de Henares, Madrid, Spain; 2 Department of Research and Development, Química Sintética S. A., c./Dulcinea s/n, 28805 Alcala de Henares, Madrid, Spain; 3 Department of Organic Chemistry, University of Alcalá, 28871 Alcalá de Henares, Madrid, Spain

**Keywords:** nucleosides, acyclovir, drug impurities, phase transfer catalysis

## Abstract

The synthesis of 9,9'-[1,2-ethanediylbis(oxymethylene)]bis-2-amino-1,9-dihydro-6*H*-purin-6-one, a minor impurity of acyclovir, is described. Starting with commercial *N*-(9-acetyl-6-oxo-1*H*-purin-2-yl)acetamide, the process uses an acid catalysed phase transfer catalysis (PTC) process to produce the selective alkylation at the 9 position of the guanine ring.

## 1. Introduction

Drug impurities, called in the literature related substances in an active pharmaceutical ingredient (API), can have a significant impact on the quality and safety of the pharmaceutical preparations. Therefore, in any API, it is necessary to study the impurity profile, and control it during the preparation of the pharmaceutical. As indicated in the ICH guidelines, any impurity, formed at a level of ≥0.10% with respect to the API, should be identified, synthesized and characterized thoroughly [1]. ICH classifies organic impurities as starting materials, by-products, intermediates, degradation products, reagents, ligands and catalysts. All of them, in addition to determine the quality and suitability of the API, are related with possible toxic episodes, or can be used to detect the synthetic method used to obtain the main product [2,3,4,5,6].

**Figure 1:**
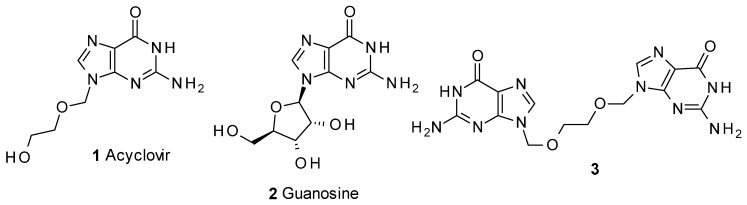
Acyclovir and related compounds.

Acyclovir (**1**, Figure 1) [7,8] is a guanosine analogue antiviral prodrug used for the treatment of herpes simplex and herpes zoster, and is one of the most commonly used antiviral drugs. Different synthetic approaches have been described to obtain this compound, many of them in patents [9,10,11,12,13,14] describing alternatives for the preparation of the product. Detailed chemical strategies and mechanisms involved have been detailed in a review [15].

Of the processes described, those involving the use of 2-benzoyloxyethoxymethylene chloride (**7**) as intermediate [9,10] can give rise to the appearance of **3** (Figure 1) as a minor impurity of **1**. Compound **3** has been cited [16] as part of an HPLC study into acyclovir-related compounds. In our case, the product was obtained in order to supply samples for analysis validation.

**Scheme 1:**
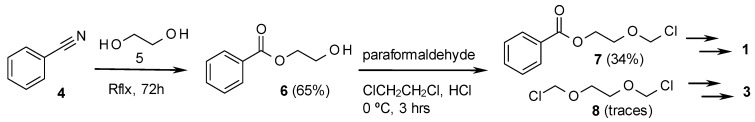
Initial steps of the synthesis of acyclovir.

The reason for the formation of **3** is outlined in Scheme 1. The synthesis of acyclovir (**1**) through the approaches outlined above requires the preparation of **7** [9,10,17] from the ester **6** [17]. Compound **6** is usually obtained by monoacylation of ethylene glycol, which is used in large excess in the process. Usually, **6** is purified by distillation, which could easily produce—particularly in large scale preparations—contaminant traces of the starting glycol. On preparing **7** with paraformaldehyde and HCl, traces of the symmetric compound **8** would be produced, and the rest of the process would yield variable amounts of **3** as a contaminant of acyclovir. This has been a common problem in the monofunctionalisation of 1,2-diols [18,19], although recently a variety of catalysts as lanthanide salts, zeolites and tin derivatives, among others, have been successfully applied to the monofunctionalisation [20]. Compound **3** is not listed as an official impurity by the European Pharmacopea [21].

## 2. Results and Discussion

**Scheme 2:**
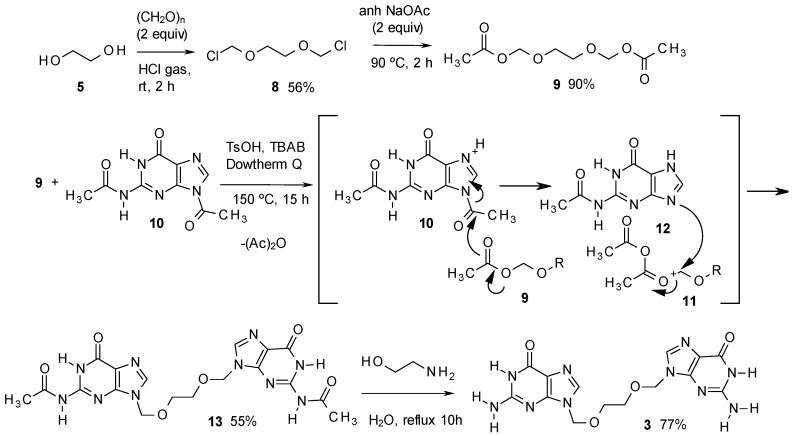
Synthesis of impurity **3**.

The method for the preparation of **3** is described in Scheme 2, and starts with ethylene glycol, converted with paraformaldehyde and hydrogen chloride without solvent, to the dichloro derivative **8** [22]. Compound **8** was subsequently converted into the diacetate **9**. This product was then treated in Dowtherm Q at 150 °C with 2,9-diacetylguanine (**10**), in the presence of TsOH as acid catalyst and tetrabutylammonium bromide (TBAB) as phase transfer catalyst. In the absence of TBAB, no transformation took place. 

The process indicated in Scheme 2 produced the diacetyl derivative **13**, yielding acetic anhydride as a subproduct. The method is based in an unusual acid catalysed PTC process described in the 70s by Landini *et al.*, who used it to convert primary alcohols into alkyl chlorides using aqueous HCl [23], to cleave ethers and esters with aqueous HBr, with conversion of the resulting aliphatic alcohols into bromides [24], or to add hydrohalogenic acids to alkenes, using aqueous solutions of the corresponding acid [25]. The method has been more recently improved by Goverdhan *et al.*, with the use of microwaves, to cleave cyclic ethers to halo alcohols, lactones to haloacids or diols to haloalcohols [26].

**Figure 2:**
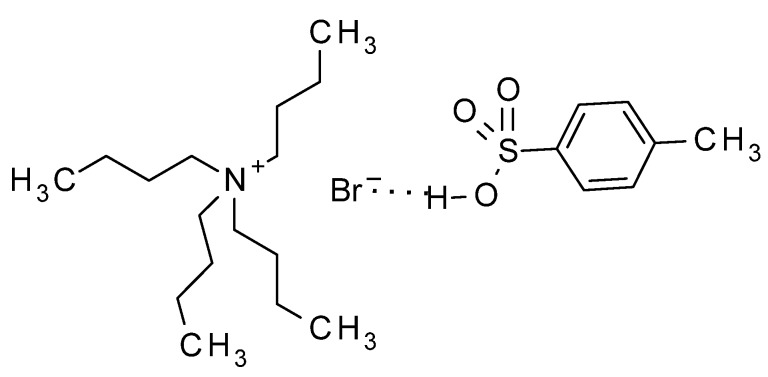
TBAB ion pair with the associated acid catalyst.

Having in mind those results, in our method, the acid, associated to the phase transfer catalyst by a hydrogen bond, as indicated by Landini, should be transferred to the organic solvent (Figure 2), thus activating the basic nitrogen of the imidazole moiety, and making the 1-acetyl group more reactive (Scheme 2). Thus, the acetyl group should be transferred to **9**, yielding the oxonium intermediate **11**, which is attacked by the basic nitrogen of **12**, displacing acetic anhydride and yielding **13** as the main product. The last step to deacylate **13**, has been extensively described for acyclovir [11], being ammonia in methanol the most common method [11,27,28], or alternatively, hot aqueous methylamine [29,30]. In our case, the best yields were obtained by adapting the methylamine procedures by treatment of **13** with 2-aminoethanol, which produced the final product **3** with the best purity and yield. The method has been scaled up for the preparation of up to 10 g of the target compound.

## 3. Experimental

### General

*N*-(9-acetyl-6-oxo-1*H*-purin-2-yl)acetamide, was obtained from Zhejiang Oma Pharmaceuticals. All other chemicals and solvents were purchased from the usual commercial sources. Melting points were determined on a Stuart Scientific SMP3 melting point apparatus using open capillary tubes and are uncorrected. IR spectra were recorded on a Perkin–Elmer FTIR 1725X spectrophotometer. ^1^H- and ^13^C-NMR spectra were recorded on either Varian Gemini (200 MHz), Unity (300 or 500 MHz) spectrometers, using TMS as internal reference. Mass data were collected on an Agilent 6210 Time-of-Flight LC/MS.

*1,2-Bis-chloromethoxyethane* (**8**) [16]. HCl gas was bubbled for 2 h into a suspension of paraformaldehyde (77.4 g, 2.58 mol) in ethylene glycol (71.9 mL, 1.29 mol). After that, the reaction mixture became transparent and two phases were observed. The mixture was poured into a separation funnel, the lower phase was separated and the upper phase was extracted with dichloromethane (70 mL). The combined organic phases were dried over anhydrous Na_2_SO_4_ and concentrated under reduced pressure. The product was isolated by distillation under reduced pressure (20 mbar) and different fractions were separated. The desired product **8** distilled between 95–110 °C as a colourless transparent liquid (119.81 g, 58% yield). ^1^H-NMR (500 MHz, CDCl_3_) ppm: δ 3.88 (s, 4H), 5.50 (s, 4H). ^13^C-NMR (125 MHz, CDCl_3_) ppm: δ 68.5 (2CH_2_), 82.6 (2CH_2_).

*1,2-Bis(diacetoxy-methyloxy)ethane* (**9**) [16]. Compound **8** (59.96 g, 0.397 mol) was placed in a 200 mL flask equipped with a mechanical stirrer. The flask was cooled with an ice-water bath and anhydrous NaOAc (67.74 g, 0.826 mol) was added in four portions over 1 h. On completion of the addition, the mixture was heated at 90 °C for 1 h 30 min and then allowed to cool to room temperature. The reaction mixture was filtered and the solid (sodium chloride) was washed with dichloromethane (3 × 70 mL). The combined organic phases were concentrated under reduced pressure. The product was purified by distillation under reduced pressure (150 °C, 20 mmHg) to give **9** as a colourless transparent liquid (73.40 g, 90% yield). IR (NaCl film): 1744 cm^−1^ (C=O). ^1^H-NMR (300 MHz, CDCl_3_) ppm: δ 2.07 (s, 3H), 3.78 (s, 2H), 5.27 (s, 2H). ^13^C-NMR (125 MHz, CDCl_3_) ppm: δ 21.0 (2CH_3_), 69.3 (2CH_2_), 89.2 (2CH_2_), 170.5 (2C).

*N-{9-[2-(2-Acetylamino-6-oxo-1,6-dihydropurin-9-ylmethoxy)-ethoxymethyl]-6-oxo-6,9-dihydro-1H-purin-2-yl}acetamide* (**11**) [16]. Compound **10** (1 g, 4.325 mmol), *p*-toluenesulfonic acid (0.02 g), tetrabutylammonium bromide (0.03 g) and Dowtherm Q (13 mL) as solvent, were placed in a three-necked flask equipped with a distillation system and a N_2_ inlet. The mixture was heated under an inert atmosphere at 110–120 °C and a solution of **9** (0.97 g, 4.7 mmol) in Dowtherm Q (1 mL) was added in three portions over 1 h. Vacuum was applied (100 mmHg) and the flask was heated at 150 °C to distil off acetic anhydride. The reaction mixture became orange and, after 15 h heating, compound **9** had been consumed. The mixture was allowed to cool to room temperature and acetone (20 mL) was added. The mixture was stirred and cooled in an ice-water bath for 1 h 30 min. A solid appeared that was filtered and washed with acetone (2 × 15 mL) to give **11** as a white solid. The solid was suspended in DMF (3 mL) and the mixture was heated to 100 °C for 30 s [31]. The solid was filtered and dried (1.05 g, 52% yield; 92% purity by HPLC; mp 301.9 °C). ^1^H-NMR (500 MHz, DMSO-d_6_) ppm: δ 2.16 (s, 6H), 3.56 (s, 4H), 5.40 (s, 4H), 8.08 (s, 2H), 11.71 (s, 2H). ^13^C-NMR (125 MHz, DMSO-d_6_) ppm: δ 23.1 (2CH_3_) + 30.0 (2CH_2_), 67.2 (2CH_2_), 72.0 (2CH_2_), 119.6 (2C), 139.4 (2CH), 147.5 + 148.2 (2C), 154.3 (2C), 172.9 (2C), 205.7 (2C). HRMS (FAB+): Calc. for C_18_H_20_N_10_O_6_ [M^+^+1] = 473.1646; found [M^+^+1] = 473.1647. 

*9,9'-[1,2-Ethanediylbis(oxymethylene)]bis-2-amino-1,9-dihydro-6H-purin-6-*one (**3**). Compound **11** (0.8 g, 1.7 mmol) was suspended in distilled water (8 mL) and ethanolamine (0.8 mL, 0.08 mol) was added. The mixture was heated under reflux for 10 h and then allowed to cool to room temperature. Aqueous HCl (10%) was added to give pH 7 and the mixture was heated at 90 °C for 15 min. The solid was filtered off and dried in a vacuum oven at 40 °C. The solid was dissolved in DMSO (8 mL) and heated to 80–90 °C for 15 min. The solution was filtered and water (10 mL) was added dropwise. The mixture was stirred at room temperature for 30 min. A white solid formed and the suspension was filtered, the residue suspended in DMSO (8 mL) and heated to 80–90 °C for 15 min. Then, the solution was filtered and water was added dropwise (~10 mL) until the liquid became cloudy. The mixture was then stirred at room temperature for 30 min. The resulting white solid was filtered and dried in a vacuum oven at 100 °C. Compound **3** was obtained as a white powder (0.51 g, 77% yield; 95% purity by HPLC, Mp > 400 °C). ^1^H-NMR (500 MHz, DMSO-d_6_) ppm: δ 3.54 (s, 4H), 5.30 (s, 4H), 6.51 (s, 4H), 7.78 (s, 2H), 10.63 (s, 2H). ^13^C-NMR (125 MHz, DMSO-d_6_) ppm: δ 40.6 (CH_2_), 56.9 (CH_2_), 67.0 (2CH_2_), 71.4 (2CH_2_), 116.0 (2C), 137.1 (2CH), 150.9 (2C), 153.3 (2CH), 156.2 (2C). HRMS (FAB+): Calc. for C_14_H_16_N_10_O_4_ [M+1] = 389.3464; found [M^+^+1] = 389.3466. Higher purity can be obtained by repeating the DMSO/water crystallisation process.

## 4. Conclusions

Validation of analytical methods of active pharmaceutical ingredients (API) requires the availability of reference samples of impurities eventually formed in the synthetic processes of the corresponding API, to be used as standards. In this case, a rare impurity of acyclovir has been obtained through an acid catalysed PTC process which ensures regioselective alkylation of 2,9-diacetylguanine (**10**).
